# Assessing the Psychological Impact of COVID-19 among College Students: An Evidence of 15 Countries

**DOI:** 10.3390/healthcare9020222

**Published:** 2021-02-17

**Authors:** Kavita Batra, Manoj Sharma, Ravi Batra, Tejinder Pal Singh, Nena Schvaneveldt

**Affiliations:** 1 Office of Research, School of Medicine, University of Nevada, Las Vegas, NV 89102, USA; 2 Department of Environmental and Occupational Health, University of Nevada, Las Vegas, NV 89119, USA; manoj.sharma@unlv.edu; 3 Department of Information Technology and Testing Center of Excellence, Coforge, Atlanta, GA 30338, USA; ravi.batra@coforgetech.com; 4 Department of Family and Preventive Medicine, Division of Public Health, School of Medicine, University of Utah, Salt Lake City, UT 84108, USA; tp.singh@utah.edu; 5 Spencer S. Eccles Health Sciences Library, University of Utah, Salt Lake City, UT 84112, USA; nena.schvaneveldt@utah.edu

**Keywords:** COVID-19, SARS-COV-2, anxiety, depression, stress, suicidal ideation, students

## Abstract

Mental health issues among college students is a leading public health concern, which seems to have been exacerbating during the COVID-19 pandemic. While previous estimates related to psychological burden among college students are available, quantitative synthesis of available data still needs to be performed. Therefore, this meta-analysis endeavors to present collective evidence discussing the psychological impact of COVID-19 among college students. Bibliographical library databases, including Embase, Medline, CINAHL, Scopus, and PsycINFO, were systematically searched for relevant studies. Titles, abstracts, and full articles were screened, and two reviewers extracted data. Heterogeneity was assessed by I^2^ statistic. The random-effects model was utilized to obtain the pooled estimates of psychological indicators among college students. Location, gender, level of severity, and quality scores were used as moderator variables for subgroup analyses. Funnel plot and Egger linear regression test was used to assess publication bias. Twenty-seven studies constituting 90,879 college students met the inclusion criteria. The results indicated 39.4% anxiety (95% CI: 28.6, 51.3; I^2^ = 99.8%; *p*-value < 0.0001) and 31.2% depression (95% CI: 19.7, 45.6; I^2^= 99.8%, *p* < 0.0001) among college students. The pooled prevalence of stress (26.0%), post-traumatic stress disorder (29.8%), and impaired sleep quality (50.5%) were also reported. College students bear a disproportionate burden of mental health problems worldwide, with females having higher anxiety and depression levels than males. This study‘’s findings underscore the need to develop appropriate public health interventions to address college students’ emotional and psychosocial needs. The policies should be reflective of demographic and socioeconomic differentials.

## 1. Introduction

By and large, college students generally experience several challenges, including starting new relationships, new life experiences, often new living situations, often an exploration of their sexual identities, usually academic pressures, need for time management, and sometimes balancing study, work, and personal life [1]. A study of college students investigating the psychological correlates found that the top concerns among this subgroup include pressure to succeed, educational performance, and post-college graduation plans [2,3]. These challenges make these students vulnerable to distress and associated negative sequelae such as depression, anxiety, insomnia, suicidal ideation, and adoption of maladaptive behaviors [1,2,3]. 

Mental health issues are alarmingly high among college students, particularly in the United States, with every eight in ten students experiencing frequent stress episodes in 2019 [4]. An eight-country study of 13,984 first-year college students under the World Health Organization’s (WHO) World Mental Health Surveys found that the lifetime and annual prevalence of suicidal ideation in this group was 32.7% and 17.2%, respectively, which correspond to the high distress levels in the students’ subgroup [5]. The likelihood of suicidal ideation increased twice following one or two traumatic events [1]. Among predictors of major depressive disorders, prior suicide plans/attempts, a history of childhood traumatic or stressful events, and family history contributed to college students’ mental adversities [6]. These data are especially relevant in the context of U.S. college students, and the proportion of affected students may vary from country to country. Nonetheless, the mental health issues of college students emerge as a critical public health concern.

Mental health problems adversely affect numerous aspects of life. For college students, academic performance is the first to be affected. A Belgian study found that mental health problems have reduced college students’ grade point average (GPA) by 0.2 to 0.3 points [7]. Depressive disorders among students are associated with cognitive impairments and real-world functioning [8]. The psychological impact among students extends further to the risk of adopting maladaptive behaviors, including binge drinking, smoking, substance abuse, overeating, risky sexual activities, dependence on social media, and sleep deprivation [8,9,10]. Stigma and embarrassment are also commonly associated with mental health problems among youth [11].

In December 2019, COVID-19 emerged as a public health threat and slowly became a worldwide pandemic, showing no curtailment signs while writing this manuscript [12]. COVID-19 has placed a considerable health burden and taxed the health care services around the world. Besides having a direct impact on physical health, it has had a severe toll on the psychological well-being of individuals due to fear, uncertainty, quarantine measures, lockdowns, social isolation, “infodemic” (or outpouring of news through various outlets, including social media), and so on [13,14,15,16]. In a study performed in India’s post-phase two lockdown period, college students had higher stress and anxiety levels than the general population [17]. Many universities have closed in-person classes, vacated dormitories, and introduced online teaching, which has led to tremendous academic stress among students [18]. The adverse psychological outcomes have been compounded for students who are already facing higher levels of distress. Loneliness and insufficient perceived social support are detrimental to mental health [19], both of which have been accentuated in the COVID-19 pandemic. A mixed-methods study done at a public college in the United States found that 71% of the respondents had higher stress and anxiety with associated stressors of fear, worry, lack of concentration, and disruption in sleep during the COVID-19 pandemic [20]. College students who have recently moved away from their families are particularly susceptible to social deprivation and feelings of loneliness [21].

Further studies on students conducted in France, Ethiopia, China, and Malaysia also point at a high negative impact on college students’ psychosocial health during the COVID-19 pandemic [22,23,24]. A study of college students in China found that the prevalence of post-traumatic stress disorder and depression rose to 2.7% and 9.0% during the COVID-19 pandemic [25]. Silva Junior et al. (2020) have published a protocol for conducting a systematic review on studying the psychological consequences of COVID-19 among young adults. However, no meta-analysis has yet been performed [26]. While the pooled estimates indicating the psychological impact of COVID-19 were reported for different population groups, including healthcare workers, the general population, and patients with pre-existing disorders, the collective evidence on college students’ mental health still needs to be quantified [17,27,28,29,30]. Against this backdrop, this study attempts to conduct a meta-analysis of peer-reviewed published studies on the burden of psychological indicators among college students following the COVID-19 pandemic.

## 2. Materials and Methods

### 2.1. Protocol Registration

The preferred reporting items for systematic reviews and meta-analyses (PRISMA) guidelines were followed for this study [31]. This study’s protocol was registered with the National Institute for Health Research (CRD42020203560), which serves as a prospective systematic review register. A detailed protocol can be found at https://www.crd.york.ac.uk/PROSPERO/display_record.php?RecordID=203560 (accessed on 16 February 2021).

### 2.2. Eligibility Criteria

We adapted the eligibility criteria used in the previous reports [27] to identify non-interventional and quantitative studies assessing the psychological impact of COVID-19 among college students. Studies were grouped according to the type of psychological morbidity observed, location (continent/country), quality score, and assessment method. Studies were included which met the following criteria: (1) use of the English language; (2) published from the inception of the pandemic to 27 July 2020; (3) utilized survey tools with good psychometric properties, and (4) full texts of the studies were available. Studies with the following characteristics were excluded: (1) Studies performed on populations other than students; (2) study designs utilizing descriptive, mixed-methods, qualitative approaches; (3) studies with unclear methodology or unvalidated survey tools; (4) studies using a language other than English; (5) studies conducted after 27 July 2020; (6) studies conducted among adolescents/students with pre-existing mental conditions, such as Attention Deficit Hyperactivity Disorder (ADHD); and (7) studies lacking the individual estimates for students.

### 2.3. Sources of Information

A search strategy was adapted from previous reports [27]. Library databases, including Medline (1946–2020), Embase (1974–2020), CINAHL (1937–2020), PsycINFO (1872–2020), and Scopus (1970–2020), were systematically searched.

### 2.4. Search Strategy

An experienced medical librarian (NS) designed the Medline search and then translated that search for use in the other databases [27]. When available, a search limit to the English language was applied, as was a publication date limit of 1 December 2019 to 27 July 2020. The search strings consisted of natural language terms and (when available) controlled vocabulary representing the concepts of “COVID-19” and “psychological outcomes.” A detailed search strategy can be found in Appendix A, Box A1.

### 2.5. Selection Process

**Figure 1:**
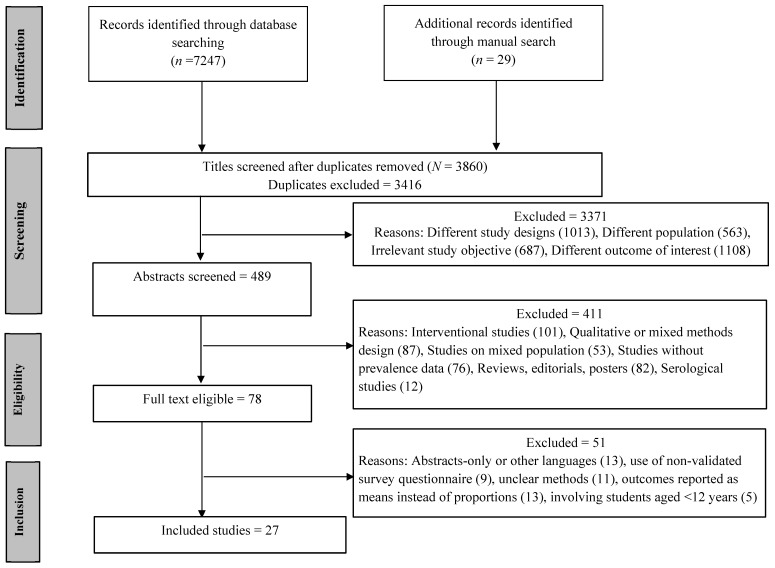
Preferred reporting items for systematic reviews and meta-analyses (PRISMA) flow diagram detailing all steps of screening with reasons for exclusion.

The search results were imported to Rayyan for the screening process. Two investigators (KB and MS) were involved in the screening of titles and abstracts to assess the articles’ relevance with the research objective (Figure 1, Identification step). During the second level of screening, KB and MS independently evaluated all potential full-text articles (Figure 1, Screening step). In case of disagreements, the consensus among reviewers was built through discussions. The included publications addressed the psychological outcomes of COVID-19 among students. If multiple studies from the same authors were found, only the most recent manuscript was included in the analysis to avoid duplicate data bias. If any data discrepancies were noted in the articles, corresponding authors were contacted for verification. 

### 2.6. Data Collection

Full-text articles were obtained for all studies that initially met the inclusion criteria. Two independent reviewers (KB and RB) abstracted all studies for potential inclusion and quality using a customized data abstraction form, resulting in an interrater agreement of 81%. Inconsistencies between reviewers were adjudicated by a third independent reviewer (MS). Information related to study authors, publication year, study location, gender distribution, number of subjects, type of survey tool with the cut-off criteria, and the proportion of subjects with positive psychological outcomes were collected in a spreadsheet. Data were reviewed twice to ensure accuracy. We also attempted to contact corresponding authors of the primary studies to verify the accuracy of data points (if needed).

### 2.7. Assessment of Risk of Bias in Primary Studies

The National Institutes of Health (NIH) quality assessment tool was utilized for the risk of bias assessment. Two reviewers (KB and MS) independently evaluated the risk of bias and assigned the quality scores based on the tool’s dictionary and guidelines (Appendix A, Table A1). The overall quality score was assigned according to the tool guidelines. In case of disagreements, the consensus among reviewers was built through discussions. 

### 2.8. Measures of Effect and Data Analysis 

The Comprehensive Meta-Analysis Package (CMA version 3.0, Englewood, NJ, USA) was utilized to compute the pooled estimates of psychological outcomes, including anxiety, depression, and other psychological indicators. The effect measure was the proportion of anxiety and depression events. The logit transformation of the proportions was used to meta-analyze the data. The Clopper–Pearson method was used to calculate exact confidence intervals for individual studies. Owing to methodologic differences across studies, a random-effects model was used to extract the pooled estimate [32]. Substantial heterogeneity was defined as I^2^ > 75% [33]. Subgroup analyses by continent (Asia vs. other), country (China vs. other), survey tool, study quality, gender, and levels of psychological outcomes were performed. Sensitivity analysis or leave-one-out analysis was also conducted to determine the impact of different weights assigned to each study on the final results. Funnel plot and Egger linear regression test statistics were utilized for publication bias [27,34]. *p*-values less than 0.05 were considered significant.

### 2.9. Assessment of Evidence

We assessed the certainty of the overall evidence based on the quality of individual studies and scientific rigor of the methodology used in each study. Two reviewers assessed the quality of the evidence and did not know each other’s decision. 

## 3. Results

### 3.1. Selection of the Dtudies 

A total of 7276 relevant records were identified following systematic and manual search (Figure 1). The titles of the remaining 3860 records (after removing 3416 duplicates) were screened, of which only 489 articles advanced to the abstract screening step. Only 78 articles were found eligible (51 articles excluded) for the full-text screening, which later reduced to 27 articles for the final review or analysis. Reasons for exclusion are listed in Figure 1.

### 3.2. Characteristics of Included Studies 

Twenty-seven studies (Appendix A, Table A2) [19,25,35,36,37,38,39,40,41,42,43,44,45,46,47,48,49,50,51,52,53,54,55,56,57,58,59] with a sample size of 90,879 students were finally assessed for generating pooled estimates. Eighteen studies were from Asia (14 from China, 1 from India, 1 from Israel, 1 from Jordan, and 1 from Saudi Arabia), seven were from Europe (two from Turkey, one from France, one from Greece, one from Italy, one from Russia and Belarus, and one from Albania), and two were from South and North America (one each). The median number of individuals across studies ranged from 66 to 44,447, with males constituting only 35% (*n* = 31,536) of the entire population. The remaining 50.4% (*n* = 45,824) of the sample constituted females. For the remaining 15% of the gender data, individual estimates for students were not provided. 

### 3.3. Risk of Bias in the Included Studies

Eleven studies were assigned good quality scores [19,25,35,38,43,44,47,48,56,58,59] and sixteen studies were identified as of medium or fair quality [36,37,39,40,41,42,45,46,49,50,51,52,53,54,55,57] (Appendix A, Table A2). The kappa statistic (inter-rater agreement) was 89.5%. 

### 3.4. Meta-Analysis

#### 3.4.1. Anxiety

**Figure 2:**
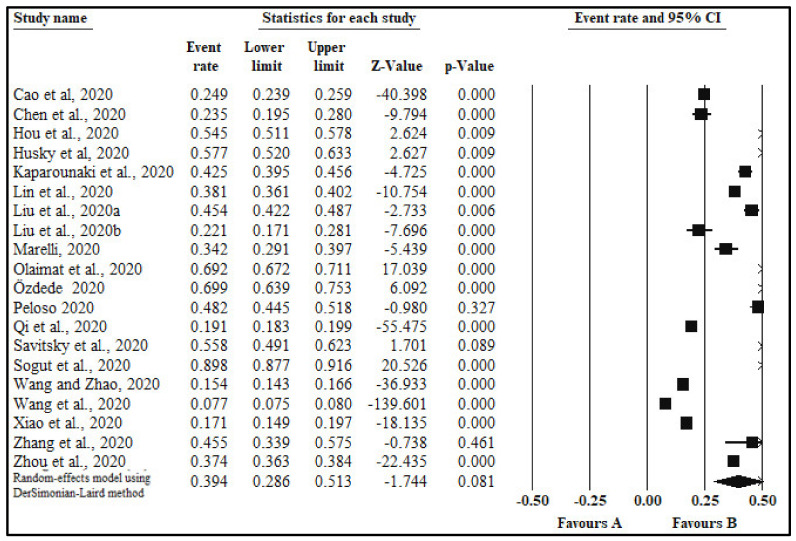
Forest plot showing pooled estimates of anxiety among students.

The pooled prevalence of anxiety in twenty studies [19,35,36,38,39,40,42,43,44,47,50,51,52,53,54,55,56,57,58,59] with a sample size 84,097 was 39.4% (95% CI: 28.6,51.3; I^2^ = 99.8%; *p*-value < 0.0001; Table 1, Figure 2). Sub-analyses by additional categorical moderators, including gender, quality of study, continent, country, type of survey tool, and anxiety level were also conducted. Results of sub-analyses are given in Table 1. 

#### 3.4.2. Depression

**Figure 3:**
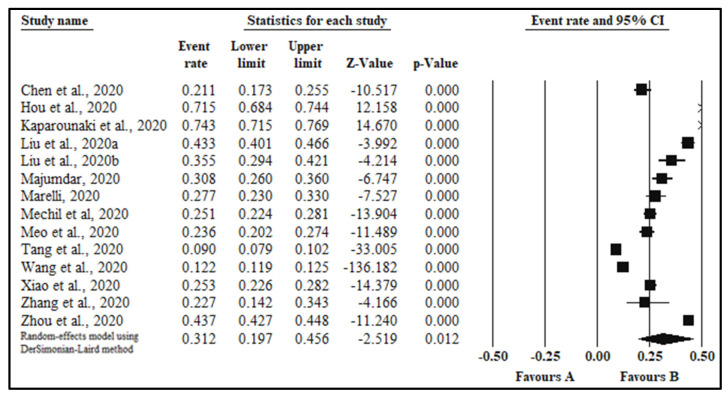
Forest plot indicating the pooled estimates of depression among students.

The pooled prevalence of depression in fourteen studies [19,25,36,38,40,43,44,46,47,48,49,57,58,59] with a sample size 61,392 was 31.2% (95% CI: 19.7,45.6; I^2^ = 99.8%, *p* < 0.0001, Table 2, Figure 3). Sub-analyses by additional categorical moderators, including gender, quality of study, continent, country, type of survey tool, and level of anxiety was also conducted (Table 2).

#### 3.4.3. Other Psychological Outcomes

The pooled prevalence of stress in three studies [39,41,58] with a sample size of 1799 was 26.0% (95% CI: 7.7,59.5; I^2^= 98.9%, *p* < 0.0001). Post-traumatic stress disorder (PTSD) in a sample of 4242 students across three studies [25,38,43] was 29.8% (95% CI:3.0, 85.4; I^2^ = 99.8%, *p* < 0.001). The overall prevalence of impaired sleep quality among three studies [46,47,58] in a sample size of 698 was 50.5% (95% CI:23.9,76.8; I^2^ = 97.6%; *p* < 0.001). Suicidal ideation was assessed in only two studies [38,40] with rates of 31.3% and 63.3% respectively. 

#### 3.4.4. Publication Bias

Except anxiety (*p* = 0.11), P values of Egger test indicate insignificant publication bias for depression (*p* = 0.17), stress (*p* = 0.68), sleep disturbances (*p* = 0.99), and PTSD (*p* = 0.78).

#### 3.4.5. Certainty of the Evidence

All primary studies were cross-sectional; therefore, the quality of the evidence would be moderate. However, most of the studies included in this analysis were of fair and good quality, which contributes to the certainty of the current meta-analysis evidence. 

## 4. Discussion

The current metanalysis included 27 studies with a sufficiently large sample of (*N* = 90,879) college students to explore psychological dimensions during the pandemic. Prior studies and a few systematic review protocols [26] investigated the association between psychological health outcomes and COVID-19, but quantitative synthesis was lacking. To our knowledge, the current meta-analysis provides the first collective evidence of the negative psychological burden of COVID-19 on the mental health of college students. This evidence is critical to inform colleges, universities, and other educational institutions in designing interventions and policies to improve college students’ mental health. Previous global evidence indicated that psychological morbidities were long-standing issues among college students even before the pandemic, with nearly 50% of mental issues starting at an early age of 14 years [60,61,62]. Globally, suicide remains among the leading causes of death among adolescents, which warrants the need to develop early interventions to address this population’s mental health and emotional needs [62]. The consequences of not addressing these concerns during the early phases of life will be dire. A lack of early intervention may lead to psychological morbidities in later life phases [62]. Regarding the pandemic, it is important to intervene early to promote post-traumatic growth among students in existing and repairing phases of the pandemic. Our findings suggest a higher prevalence of anxiety (39.4%), depression (31.2%), and stress (26.0%) than those reported in the pre-pandemic period with 22.1% anxiety, 19.7% depression, and 13.4% stress [60,61,62]. Corollaries associated with COVID-19, including uncertainty and fear, exert an additional driving force to explain these rising trends [24]. The timeline to graduation, sudden transition to virtual learning, quality and logistics of internships, and post-graduation plans are all in uncertainty, causing significant distress among college students [24,52]. Association of other contributing factors, such as compliance to the new rules, propagation of ambiguous messages through media, and lack of scientific understanding, need to be explored fully to design a holistic public health approach to address mental health challenges among college students [60,62].

Additionally, young people like to socialize and indulge in parties and celebrations, which have been restricted in pandemic times, adding to their frustration levels [52,53,57]. Some students who receive counseling services have not been able to receive such support. Many students who work part-time jobs have lost their employment (voluntarily or employer initiated) during COVID-19, causing financial distress [24,52,53,57]. According to a study of 69,054 French students, nearly 42.8% of students reported having at least one negative mental health outcome; of those, only 12.4% sought assistance from healthcare professionals [24]. The stigma associated with seeking mental health support has been cited as a primary factor of underreported mental health issues among adolescents [62]. Among risk factors, the female gender is associated mainly with psychosocial health [24,53]. Females were twice as likely as males to experience mental health issues [24]. Our study found a significant gender gap in psychological morbidities. Females had significantly higher anxiety levels (34.6% vs. 22.9%) and depression (32.4% vs. 26.0%) than males. This finding was consistent with previous studies [24,63,64]. The gender differences may be attributed to a higher prevalence of pre-existing mental health conditions among females than males, complicated by introversion, higher sensitivity to traumatic events, and other factors, including hormonal imbalances and genetic vulnerability, and a higher mental health stigma among men [64,65,66]. Additional evidence reported that it is likely that mental health issues among men are underreported because of their tendency not to seek help from others [67].

We found a wider variation while making country comparisons. Anxiety and depression reported out of Asian countries were lower compared to other countries. Traditional close-knit family systems in Asia can be a protecting factor overriding one significant risk factor of social isolation, which has shown to contribute to increased risk of mental health issues [66]. Additionally, Asian countries, especially China and India, have traditional medicine with products and services widely available that are acceptable, affordable, and culturally appropriate. Most importantly, these have been adopted by the various Asian countries’ health care systems [68]. However, the efficacy of traditional medicine has not been fully proven in counteracting mental health problems.

### 4.1. Strengths and Limitations

This meta-analysis is the first to assess the psychological impact of COVID-19 among students. It is urgent and essential to know the global scope of the issue. This population group is already facing a disproportionate burden of psychological morbidities even before the pandemic. This study also has some limitations. First, the self-reporting nature of the data collected by the studies in our meta-analysis might not be an accurate representation of the clinical diagnosis of the psychological illness. Second, sampling bias may exist because nearly 66.6% (18 out of 27) of the studies were conducted in Asia and predominantly China (51.8%; 14 out of 27). The larger pool of studies from China may presumably be due to the greater interest of the Chinese researchers in unfolding the epidemiology of COVID-19, as China was the first country to be affected by COVID-19. Other countries might have other research priorities prior to the pandemic inception, which occurred two months following the pandemic emergence in China. Third, all studies included in this meta-analysis were cross-sectional, which only account for prevailing circumstances, thereby lacking a longitudinal aspect to encounter temporality. Fourth, the studies included were only published in the English language, which might have introduced a language bias. Last, most of the studies included in this meta-analysis did not provide the year-wise, program (undergraduate/postgraduate), and type of course (e.g. STEM vs. non-STEM) stratifications of the students, which restricted our ability to determine differences in psychological morbidities among these groups. Future studies can be designed to account for differences in psychological outcomes across different groups of students to design a more targeted interventional approach. 

### 4.2. Implications for Practice

This study advocates for designing and implementing appropriate interventions or programs to promote the mental health of students. The new policies and interventions will need to address gender differentials, such as designing tailored interventions for girls to address their specific needs. The use of telehealth has also been expanded in COVID-19, which can be used to offer remote counseling interventions across school or college campuses. Online implementation of mental health programs should be emphasized in lower or middle-income countries, which was reported to be a neglected field despite having good internet use [68]. Regular counseling centers for in-person visits across campuses with limited access to technology can also be beneficial. Besides, efforts should be directed towards increasing the quality of mental health services provided to the students. Mental health services provided by trained staff are improving. However, there are some gaps to be filled. According to the Association for College and College Counseling Center Directors (AUCCCD), comprising counseling directors of educational institutions from the United States, Canada, Europe, the Middle East, Asia, and Australia, one in five centers on their campus were reported to be lacking the optimum quality of mental health services [61,62]. The guided ways of stress management as implemented in certain universities in the US can be tailored towards a more comprehensive virtual delivery during the times of COVID-19. The American Council on Education advisory for the leadership ensures readiness of campuses for handling the increased burden on students’ mental health. This involves regularly performing the needs assessment of college students from diverse backgrounds to design prospective policies and interventions. Healthy Minds Study or the American College Health Association-National College Health Assessment are examples that can be launched campus-wide to collect data for assessment and targeted intervention development. 

## 5. Conclusions

College students bear a disproportionate burden of mental health problems worldwide, with females having higher anxiety and depression levels than males. This study’s findings underscore the need to develop appropriate public health interventions to address adolescents’ emotional, psychological, and social needs. The policies should be reflective of demographic and socioeconomic differentials. 

## Figures and Tables

**Table 1:** Pooled estimates of anxiety by categorical moderator variables (subgroup analyses).

Overall		Number of Studies	Proportion (%)	95% CI	I^2^	*p*-Value	References
Anxiety prevalence		20	34.4%	29.5,39.7	99.1%	<0.0001	[19,35,36,38,39,40,42,43,44,47,50,51,52,53,54,55,56,57,58,59]
**Subgroup Analysis**							
**Categories**	**Subgroups**	**Number of Studies**	**Proportion (%)**	**95% CI**	**I^2^**	***p*-Value**	**References**
Quality	Good	9	29.3	16.8,45.8	99.8%	<0.0001	[19,35,38,43,44,47,56,58,59]
	Medium	11	48.4	33.0,64.1	99.6%	<0.0001	[36,39,40,42,50,51,52,53,54,55,57]
Continents	Asia	13	30.4	20.0,43.4	99.8%	<0.0001	[19,35,36,38,42,44,50,53,54,56,57,58,59]
	Other	7	57.5	38.6,74.4	98.8%	<0.0001	[39,40,43,47,51,52,55].
Countries	China	11	25.5	16.7,36.9	99.8%	<0.0001	[19,35,36,38,42,44,53,56,57,58,59]
	Other	9	58.7	44.0,72.0	98.7%	<0.0001	[39,40,43,47,50,51,52,54,55]
Assessment	GAD	8	33.0	18.1,52.3	99.4%	<0.0001	[35,38,43,44,53,54,57,59]
	Other	12	43.9	28.9,60.1	99.8%	<0.0001	[19,36,39,40,42,47,50,51,52,55,56,58]
Gender	Female	5	34.6	20.5,52.0	99.0%	<0.0001	[54,56,57,58,59]
	Male	5	22.9	36.3,52.5	98.3%	<0.0001	[54,56,57,58,59]
Level of Anxiety	Mild	7	73.7	63.8,81.7	96.9	<0.0001	[44,53,54,55,56,57,59]
	Moderate	7	23.1	16.2,31.8	97.7	<0.0001	[44,53,54,55,56,57,59]
	Severe	7	7.0	4.8,11.3	92.3	<0.0001	[44,53,54,55,56,57,59]

GAD: Generalized Anxiety Disorder.

**Table 2:** Pooled estimates of depression by categorical moderator variables (subgroup analyses).

Overall		Number of Studies	Proportion (%)	95% CI	I^2^	*p*-Value	References
Depression prevalence		14	31.2	19.7,45.6	99.8%	<0.0001	[19,25,36,38,40,43,44,46,47,48,49,57,58,59]
**Subgroup Analysis**							
**Categories**	**Subgroups**	**Number of Studies**	**Proportion (%)**	**95% CI**	**I^2^**	***p*-Value**	**References**
Quality	Good	9	29.7	16.4,47.7	99.8%	<0.0001	[19,25,38,43,44,47,48,58,59]
	Medium	5	34.0	15.6,59.0	99.3%	<0.0001	[36,40,46,49,57]
Continents	Asia	10	27.3	15.6,43.2	99.8%	<0.0001	[19,35,36,38,42,44,50,53,54,56,57,58,59]
	Other	4	42.2	19.3,69.1	99.3%	<0.0001	[40,43,47,48]
Countries	China	8	27.3	14.4,45.6	99.8%	<0.0001	[19,25,36,38,44,57,58,59]
	Other	6	36.8	18.8,59.5	99.1%	<0.0001	[40,43,46,47,48,49]
Assessment	PHQ	7	33.9	18.3,53.9	99.5%	<0.0001	[25,38,43,44,48,57,59]
	Other	7	28.7	14.9,48.0	99.6%	<0.0001	[19,36,40,46,47,49,58]
Gender	Female	5	32.4	20.0,44.8	96.4%	<0.0001	[44,49,57,58,59]
	Male	5	26.0	16.9,37.8	95.5%	<0.0001	[44,49,57,58,59]
Level of Anxiety	Mild	4	55.6	35.8,73.7	90.5%	<0.0001	[44,48,57,59]
	Moderate	4	30.4	17.5,47.5	97.4%	<0.0001	[44,48,57,59]
	Severe	4	16.1	8.2,29.3	96.9%	<0.0001	[44,48,57,59]

PHQ: Patient Health Questionnaire.

## Data Availability

Data are contained within the article or Appendix A.

## Appendix A

Box A1 Detailed search strategy (executed 27 July 2020) for the identfication of records discussing the psychological impact of COVID-19 among college students Database: Ovid MEDLINE(R) and Epub Ahead of Print, In-Process & Other Non-Indexed Citations and Daily <1946 to 27 July 2020> Search Strategy: ------------------------------------------------------------------------------------------------------------------- 1  (2019nCoV or 2019-nCoV or coronavirus or coronavirinae or (corona adj3 (virinae or virus)) or “Corona virinae19” or “Corona virinae2019” or “corona virus19” or “corona virus2019” or Coronavirinae19 or Coronavirinae2019 or coronavirus19 or coronavirus2019 or covid19 or COVID-19 or SARS-CoV-2 or “Severe Acute Respiratory Syndrome Corona virus 2” or “Severe Acute Respiratory Syndrome Coronavirus 2”).ti,ab,kw. [covid-19 keywords] (46270) 2  coronavirus/ or Coronavirus Infections/ [covid-19 MeSH] (19104) 3  or/1-2 [covid-19 set] (48733) 4  mental health/ or mental fatigue/ or Affective Symptoms/ or psychological distress/ [Mental health MeSH] (53257) 5  (emotional disturbanc* or affective symptom* or Alexithymia* or ((mental or psychological) adj3 (fatigue or health or status or distress or well-being)) or psychosocial).ti,ab,kw. [mental health keywords] (283768) 6  or/4-5 [mental health set] (305532) 7  Stress, Psychological/ or occupational stress/ or compassion fatigue/ or burnout, psychological/ or burnout, professional/ [stress MeSH] (131108) 8  (stress* or "adaptation syndrome" or (caregiver adj4 (burden or fatigue)) or "compassion fatigue" or "reality shock" or "social defeat").ti,ab,kw. [stress keywords] (842732) 9  or/7-8 [stress set] (897231) 10  Depression/ or anhedonia/ [depression MeSH] (119688) 11  (depression or depressed or anhedonia or dysphoria or dysthymia or melancholia or sadness).ti,ab,kw. [depression keywords] (404119) 12  or/10-11 [depression set] (436174) 13  anxiety/ or catastrophization/ [anxiety MeSH] (81955) 14  (anxiety or Catastrophiz* or hypervigilan* or nervousness).ti,ab,kw. [anxiety keywords] (195877) 15  or/13-14 [anxiety set] (218337) 16  "Sleep Initiation and Maintenance Disorders"/ [insomnia MeSH] (13134) 17  (drowsiness or dyssomnia* or hypersomnia* or insomnia* or parasomnia* or sleepless* or sleepwalk* or somnambul* or somnolen* or sopor or (sleep adj5 (disorder* or disturbance* or fragmented or debt or depriv* or walk*))).ti,ab,kw. [insomnia keywords] (77668) 18  or/16-17 [insomnia set] (80743) 19  or/6,9,12,15,18 [psychosocial outcomes set] (1621212) 20  and/3,19 [final set] (2483) 21  limit 20 to yr=“2019 -Current” (2313) 22  limit 21 to english language (2221)

**Table A1:** Methodological quality assessment of included studies using the National Institutes of Health (NIH) tool.

Author/Year	Q1	Q2	Q3	Q4	Q5	Q6	Q7	Q8	Q9	Q10	Q11	Q12	Q13	Q14	Final Quality Score	Rating
Cao et al., 2020 [35]	Y	Y	Y	Y	NA	Y	N	N	Y	NA	Y	NA	NA	N	**7**	**Good**
Chen et al., 2020 [36]	Y	Y	N	N	NA	Y	N	N	Y	NA	Y	NA	NA	N	**5**	**Fair**
Gritsenko et al., 2020 [37]	Y	Y	N	N	NA	Y	N	N	Y	NA	Y	NA	NA	N	**5**	**Fair**
Hou et al., 2020 [38]	Y	Y	N	Y	NA	Y	Y	Y	Y	NA	N	NA	NA	N	**7**	**Good**
Husky et al., 2020 [39]	Y	Y	N	N	N	Y	N	N	Y	NA	Y	NA	NA	N	**5**	**Fair**
Kaparounaki et al., 2020 [40]	Y	Y	NR	N	NA	Y	N	N	Y	NA	NA	NA	NA	N	**4**	**Fair**
Li et al., 2020 [41]	Y	Y	Y	N	NA	Y	N	N	Y	NA	Y	NA	NA	N	**6**	**Fair**
Lin et al., 2020 [42]	Y	Y	NR	N	N	Y	Y	N	Y	NA	Y	NA	NA	N	**6**	**Fair**
Liu et al., 2020 [43]	Y	Y	Y	N	N	Y	N	N	Y	NA	Y	NA	NA	N	**6**	**Fair**
Liu et a., 2020 [44]	Y	Y	Y	Y	N	Y	N	N	Y	NA	Y	NA	NA	N	**7**	**Good**
Liu et al., 2020 [45]	Y	Y	NR	N	N	Y	N	Y	Y	NA	Y	NA	NA	N	**6**	**Fair**
Majumdar et al., 2020 [46]	Y	Y	N	N	N	Y	N	Y	NR	NA	Y	NA	NA	N	**5**	**Fair**
**Author/Year**	**Q1**	**Q2**	**Q3**	**Q4**	**Q5**	**Q6**	**Q7**	**Q8**	**Q9**	**Q10**	**Q11**	**Q12**	**Q13**	**Q14**	**Final quality score**	**Rating**
Marelli et al., 2020 [47]	Y	Y	N	N	Y	Y	Y	N	Y	NA	Y	NA	NA	N	**7**	**Good**
Mechil et al., 2020 [48]	Y	Y	NR	N	Y	Y	Y	N	Y	NA	Y	NA	NA	N	**7**	**Good**
Meo et al., 2020 [49]	Y	Y	Y	N	N	Y	N	N	Y	NA	Y	NA	NA	N	**6**	**Fair**
Olaimat et al., 2020 [50]	Y	Y	Y	N	N	Y	N	N	Y	NA	Y	NA	NA	N	**6**	**Fair**
Ozdede et al., 2020 [51]	Y	Y	Y	N	N	Y	N	N	Y	NA	Y	NA	NA	N	**6**	**Fair**
Peloso et al., 2020 [52]	Y	Y	Y	N	N	Y	N	N	Y	NA	Y	NA	NA	N	**6**	**Fair**
Qi et al., 2020 [53]	Y	Y	N	N	N	Y	Y	N	Y	NA	Y	NA	NA	N	**6**	**Fair**
Savitsky et al., 2020 [54]	Y	Y	Y	N	N	Y	N	N	Y	NA	Y	NA	NA	N	**6**	**Fair**
Sogut et al., 2020 [55]	Y	Y	NR	N	N	Y	Y	N	Y	NA	Y	NA	NA	N	**6**	**Fair**
Tang et al., 2020 [25]	Y	Y	NR	Y	Y	Y	Y	N	Y	NA	Y	NA	NA	N	**8**	**Good**
Wang and Zhao et al., 2020 [56]	Y	Y	NR	N	Y	Y	Y	N	Y	NA	Y	NA	NA	N	**7**	**Good**
Wang et al., 2020 [19]	Y	Y	Y	N	N	Y	Y	Y	Y	NA	Y	NA	NA	N	**8**	**Good**
Xiao et al., 2020 [57]	Y	Y	NR	Y	N	Y	Y	N	Y	NA	N	NA	NA	N	**6**	**Fair**
Zhang et al., 2020 [58]	Y	Y	NR	N	Y	Y	Y	N	Y	NA	Y	NA	NA	N	**7**	**Good**
Zhou et al., 2020 [59]	Y	Y	Y	N	Y	Y	Y	N	Y	NA	Y	NA	NA	N	**8**	**Good**

Y: Yes, b. N: No, c. NR: Not reported, d. NA: Not applicable; Q1. Clarity of research question; Q2. Detailed description of population; Q3. Participation rate of eligible participants (at least 50%); Q4. Clarity in the inclusion and exclusion criteria; Q5. Sample size justification, power description, or variance and effect estimates; Q6. Temporality of exposure and outcome; Q7. Sufficient timeframe; Q8. Levels of exposure; Q9. Description of independent variables; Q10. Frequency of exposure assessment; Q11. Description of dependent variables; Q12. Blinding; Q13. Loss to follow-up (response rate) after baseline 20% or less; Q14. Measurement of key potential confounding variables and statistical adjustment for their impact on the relationship between exposure(s) and outcome(s); Rating—Good, Fair or Poor: Good = (7–9 yes); fair = (4–6 yes).

**Table A2:** Data summarization of the included studies.

Author/Year [Reference #]	Sample Size	Quality Score	Country	Male (%)	Survey Tool	Outcomes (%)	
						(*n*)	
						Anxiety	Depression
Cao et al, 2020 [35]	7143	7	China	30	GAD7	24.9	NA
						−1776	
Chen et al., 2020 [36]	383	5	China	NA	DSRS-C	23.5	21.2
					SCARED	−90	−81
Gritsenko et al., 2020 [37]	939	5	Russia and Belarus	19	FCV-19S	NA	NA
Hou et al., 2020 [38]	859	7	China	61	PHQ9	54.5	71.5
					GAD7	−468	−614
					IESR		
Husky et al., 2020 [39]	291	5	France	25	World Mental Health International College Student Survey	57.7	NA
						−168	
Kaparounaki et al., 2020 [40]	1000	4	Greece	31	STAI	42.5	74.3
					CES-D	−425	−743
					RASS		
Li et al., 2020 [41]	1442	6	China	NA	K6	NA	NA
					IESR		
Lin et al., 2020 [42]	2086	6	China	NA	STAI	38.1	NA
						−795	
Liu et al., 2020 [43]	898	6	USA	14	PHQ8	45.4	43.3
					GAD7	−408	−389
Liu et al., 2020 [44]	217	7	China	41	PHQ9	22.1	35.5
					GAD7	−48	−77
Liu et al., 2020 [45]	198	6	China	34	SSS	NA	NA
**Author/Year**	**Sample Size**		**Country**	**Male (%)**	**Survey Tool**	**Outcomes (%)**	**Author/Year**
						**(*n*)**	
						**Anxiety**	**Depression**
Majumdar et al., 2020 [46]	325	5	India	39	CES-D	NA	30.77
							−100
Marelli et al., 2020 [47]	307	7	Italy	25	BAI	34.3	27.8
					BDI-II	−105	−85
					PSQI		
					ISI		
Mechil et al, 2020 [48]	863	7	Albania	11	PHQ9	NA	25.2
							−217
Meo et al., 2020 [49]	530	6	Saudi Arabia	45	Stress Allied Queries	NA	23.6
							−125
Olaimat et al., 2020 [50]	2083	6	Jordan	25	NA	69.2	NA
						−1441	
Özdede et al., 2020 [51]	249	6	Turkey	38	STAI	69.9	NA
						−174	
Peloso et al., 2020 [52]	704	6	Brazil (South America)	20	NA	48.2	NA
						−339	
Qi et al., 2020 [53]	9554	6	China	NA	GAD7	19	NA
						−1814	
Savitsky et al., 2020 [54]	215	6	Israel	12	GAD7	55.9	NA
						−120	
Sogut et al., 2020 [55]	972	6	Turkey	0	BAI	−873	NA
Tang et al., 2020 [25]	2485	8	China	39	PHQ9	NA	9
							−224
Wang and Zhao, 2020 [56]	3611	7	China	40	SAS	−557	NA
**Author/Year**	**Sample Size**		**Country**	**Male (%)**	**Survey Tool**	**Outcomes (%)**	**Author/Year**
						**(n)**	
						**Anxiety**	**Depression**
Wang et al., 2020 [19]	44447	8	China	45	SAS	7.7	12.2
					CES-D	−3422	−5422
Xiao et al., 2020 [57]	933	6	China	30	GAD7	17.1	25.3
					PHQ9	−160	−236
Zhang et al., 2020 [58]	66	7	China	38	DASS21	−30	−15
					PSQI		
Zhou et al., 2020 [59]	8079	8	China	46	PHQ-9	37.4	43.7
					GAD-7	−3020	−3533
